# Editorial: Protein-Material Interfaces: Fundamentals and Applications

**DOI:** 10.3389/fmolb.2022.864005

**Published:** 2022-03-18

**Authors:** Prashanth Asuri, Greta Faccio

**Affiliations:** ^1^ Department of Bioengineering, Santa Clara University, Santa Clara, CA, United States; ^2^ Independent Scientist, St. Gallen, Switzerland

**Keywords:** protein hybrids, extracellular vesicles (EVs), FRET—förster resonance energy transfer, actin bundles, protein-material interfaces

The small-scale world is no stranger to difficult-to-control interactions and complex environments. Not surprisingly, researchers have been highly invested in understanding the interactions of proteins among themselves and with other biomolecules and materials. Of particular interest is the effect of macromolecular crowding and confinement on protein interactions and behavior. These topics have been steadily gaining attention; a brief search in the PubMEd database with keywords “protein interaction” retrieves more than 4,000 hits per year since 2013. This research topic aimed to highlight fundamental and applied works that described the preparation and characterization of protein-protein and protein-material complexes. Works published as a part of the research topic reflect a high degree of interest and collaboration of the modern scientific world in this area, with contributions from the United States, Germany, Italy, and Spain, as the results of these works could have great implications for the biotechnology and healthcare industries.

A common theme throughout the contributing articles in the research topic is the investigation and application of protein hybrids (including genetic fusions, linker constructs, and protein bundles). The articles leverage the protein hybrids in a variety of ways, thus displaying the versatility and broad application of such synthesized or naturally-formed protein assemblies. The article from the Lu lab reports the development of a dual-reporter system by genetically fusing transmembrane proteins associated with extracellular vesicles (EVs) with a fluorescent protein (GFP) and an affinity peptide (6xHis) (Levy et al.). Using the transmembrane proteins as a stable anchor, this system enables both live-cell imaging (using the GFP) and affinity purification of EVs (using the 6xHis tag). The development of such multifunctional molecular tools provides researchers with the means to gain insight into the biology of extracellular vesicles, as well as to design and engineer EVs for drug delivery, therapy, and disease diagnosis. Stanzione et al. also developed fusion proteins using genetic engineering techniques. In their work, they demonstrate the utility of combining the functionality of single-chain fragment variables of antibodies with the adhesive properties of a fungal hydrophobin to develop detection platforms for the recognition of marine toxins that cause food poisoning. Their work is of particular interest as they demonstrate two different detection modalities: optical and electrochemical, which underscores the versatility of their platform. Furthermore, the sensitivity of the devices reported in the study is comparable to those already reported in the literature. Heikal et al. report the development of a simple approach using the fluorescence fluctuations and molecular brightness for the Förster resonance energy transfer (FRET) analysis of genetically encoded donor-linker-acceptor constructs (mTurquoise2.1–linker–mCitrine) at the single-molecule level Kay et al.. Their experiments support the hypothesis that the molecular brightness of the freely diffusing donor in the presence of the acceptor is lower than that of the donor alone due to FRET. The proposed approach would require a low expression level when used in living cells, thus minimizing potential interference with the cell machinery, and limit the need for ensemble averaging that is present in other methods. Finally, Castaneda et al. report on the independent and combined roles of cations and macromolecular crowding on actin bundle mechanics and organization at the nanoscale. By varying the concentrations of the cations and macromolecular crowders, their analyses help establish an understanding of how the modulus and filament packing are dependent on the conditions typically observed in crowded intracellular environments.

Another common theme among the articles (albeit not entirely surprising) is the use of sophisticated instrumentation and techniques. Various techniques offer complementary information towards a better understanding of the proteins and their interactions with target materials and in crowded environments. The articles leverage atomic force microscopy (AFM) to determine the size and mechanical properties of actin bundles Castaneda et al., single-detector fluorescence auto-correlation spectroscopy (FCS) to investigate the FRET of fluorescent proteins at the single-molecule level Kay et al., fluorescence and confocal microscopy to study extracellular vesicles in mammalian cell cultures Levy et al., and Western blots, ELISA, and even electrochemical approaches Stanzione et al. to study fusion proteins.

In summary, articles published in this research topic probe various aspects of the protein-material interface. One aspect, which is essential to applying knowledge of proteins in complex environments, is the ability to form protein hybrids. Protein hybrids and their ability to be engineered can have a great impact on medicine by providing a novel method to detect a disease state or deliver a therapeutic. Another topic broached in these works is the use of instruments and techniques to better understand protein interactions with themselves, their target, and their environment. This fundamental knowledge is essential to better guide current and future collaborations among scientists so the characterization of protein behavior in different environments can be achieved and leveraged for a wide variety of applications across numerous fields.

